# Preserving privacy in surgical video analysis using a deep learning classifier to identify out-of-body scenes in endoscopic videos

**DOI:** 10.1038/s41598-023-36453-1

**Published:** 2023-06-07

**Authors:** Joël L. Lavanchy, Armine Vardazaryan, Pietro Mascagni, AI4SafeChole Consortium, Giovanni Guglielmo Laracca, Ludovica Guerriero, Andrea Spota, Claudio Fiorillo, Giuseppe Quero, Segio Alfieri, Ludovica Baldari, Elisa Cassinotti, Luigi Boni, Diego Cuccurullo, Guido Costamagna, Bernard Dallemagne, Didier Mutter, Nicolas Padoy

**Affiliations:** 1 IHU Strasbourg, 1 Place de l’Hôpital, 67091 Strasbourg Cedex, France; 2 Department of Visceral Surgery and Medicine, Inselspital, Bern University Hospital, University of Bern, Bern, Switzerland; 3 ICube, University of Strasbourg, CNRS, Strasbourg, France; 4 Fondazione Policlinico Universitario Agostino Gemelli IRCCS, Rome, Italy; 5 University Hospital of Strasbourg, Strasbourg, France; 6 Division of Surgery, Clarunis–University Center for Gastrointestinal and Liver Diseases, St Clara and University Hospital of Basel, Basel, Switzerland; 7 Institute for Research Against Digestive Cancer (IRCAD), Strasbourg, France; 8 Sant’Andrea Hospital, Sapienza University, Rome, Italy; 9 Monaldi Hospital, AORN dei Colli, Naples, Italy; 10 Fondazione IRCCS Ca’ Granda Ospedale Maggiore Policlinico di Milano, University of Milan, Milan, Italy; 11 Policlinico Universitario Agostino Gemelli IRCCS, Rome, Italy

**Keywords:** Translational research, Computer science

## Abstract

Surgical video analysis facilitates education and research. However, video recordings of endoscopic surgeries can contain privacy-sensitive information, especially if the endoscopic camera is moved out of the body of patients and out-of-body scenes are recorded. Therefore, identification of out-of-body scenes in endoscopic videos is of major importance to preserve the privacy of patients and operating room staff. This study developed and validated a deep learning model for the identification of out-of-body images in endoscopic videos. The model was trained and evaluated on an internal dataset of 12 different types of laparoscopic and robotic surgeries and was externally validated on two independent multicentric test datasets of laparoscopic gastric bypass and cholecystectomy surgeries. Model performance was evaluated compared to human ground truth annotations measuring the receiver operating characteristic area under the curve (ROC AUC). The internal dataset consisting of 356,267 images from 48 videos and the two multicentric test datasets consisting of 54,385 and 58,349 images from 10 and 20 videos, respectively, were annotated. The model identified out-of-body images with 99.97% ROC AUC on the internal test dataset. Mean ± standard deviation ROC AUC on the multicentric gastric bypass dataset was 99.94 ± 0.07% and 99.71 ± 0.40% on the multicentric cholecystectomy dataset, respectively. The model can reliably identify out-of-body images in endoscopic videos and is publicly shared. This facilitates privacy preservation in surgical video analysis.

## Introduction

“And whatsoever I shall see or hear in the course of my profession, […] if it be what should not be published abroad, I will never divulge, holding such things to be holy secrets.”^1^

Hippocratic Oath

Surgical video analysis facilitates education (review of critical situations and individualized feedback)^2,3^, credentialing (video-based assessment)^4^ and research (standardization of surgical technique in multicenter trials^5^, surgical skill assessment)^6,7^. Despite its increasing use, the full potential of surgical video analysis has not been leveraged so far, as manual case review is time-consuming, costly, needs expert knowledge and raises privacy concerns.

Therefore, surgical data science approaches have been adopted recently to automate surgical video analysis. Artificial intelligence (AI) models have been trained to recognize phases of an intervention^8–10^, tools^8,11^ and actions^12^ in surgical videos. This allows for down-stream applications like the estimation of the remaining surgery duration^13^, automated documentation of critical events^14^, assessment of surgical skill^15^ and safety check-point achievement^16^, or intraoperative guidance^17^.

AI will continue to reduce the costs and time constraints of experts reviewing surgical videos. However, the privacy concerns regarding the recording, storing, handling, and publication of patient video data have not been extensively addressed so far. The physician–patient privilege originating from the Hippocratic Oath, protects medical data and the identity of patients from legal inquiry. A breach of medical confidentiality by medical staff is prosecutable in most countries. Endoscopic videos which are recorded while the patient is under narcosis in the operating room (OR) are particularly sensitive. They often contain scenes of the OR that could potentially reveal sensitive information such as the identity of patients or OR staff. Moreover, if clocks or calendars present in the room are captured in the video, the time or date of the respective intervention can be identified. Information about the date and time of an operation, facilitates identification of the patient undergoing surgery. These scenes recorded outside of the patient’s body are referred to as out-of-body scenes. If video recording has already been started before the endoscope is introduced into the patient, has not been stopped after the surgery was terminated or every time the endoscope is cleaned during surgery, out-of-body scenes are captured.

Recent developments in computer vision and deep learning are fueled by large-scale and publicly available datasets. In contrast, medical applications of deep learning are often limited by small-size and restricted datasets. Deidentification of endoscopic video by blurring or deleting out-of-body scenes enables recording, storing, handling, and publishing of surgical videos without the risk of a medical confidentiality breach.

This article reports the development and validation of a deep learning-based image classifier to identify out-of-body scenes in endoscopic videos, called Out-of-Body Network (OoBNet). OoBNet enables privacy protection of patients and OR staff through automated recognition of out-of-body scenes in endoscopic videos. External validation of OoBNet is performed on two independent multicentric datasets of laparoscopic Roux-en-Y gastric bypass and laparoscopic cholecystectomy surgeries. The trained model and an executable application of OoBNet are published, to provide an easy-to-use tool for surgeons, data scientists and hospital administrative staff to anonymize endoscopic videos.

## Methods

### Datasets

The dataset used for the development of OoBNet was created from surgeries recorded at the University Hospital of Strasbourg, France^18^. Four video recordings for each of the following endoscopic procedures were arbitrarily selected: Laparoscopic Nissen fundoplication, Roux-en-Y gastric bypass, sleeve gastrectomy, hepatic surgery, pancreatic surgery, cholecystectomy, sigmoidectomy, eventration, adrenalectomy, hernia surgery, robotic Roux-en-Y gastric bypass, and robotic sleeve gastrectomy. The dataset containing 48 videos was split into training-, validation-, and test-set including 2, 1 and 1 videos of each procedure, respectively.

External validation of the model was done on a random sample of 5 videos from 6 centers and two independent multicentric datasets. (1) A dataset of 140 laparoscopic Roux-en-Y gastric bypass videos from the University Hospital of Strasbourg, France and Inselspital, Bern University Hospital, Switzerland^19^. (2) A dataset of 174 laparoscopic cholecystectomy videos from four Italian centers: Policlinico Universitario Agostino Gemelli, Rome; Azienda Ospedaliero-Universitaria Sant’Andrea, Rome; Fondazione IRCCS Ca' Granda Ospedale Maggiore Policlinico, Milan; and Monaldi Hospital, Naples. This dataset was collected for the multicentric validation of EndoDigest, a computer vision platform for video documentation of the critical view of safety (CVS)^20^.

**Figure 1:**
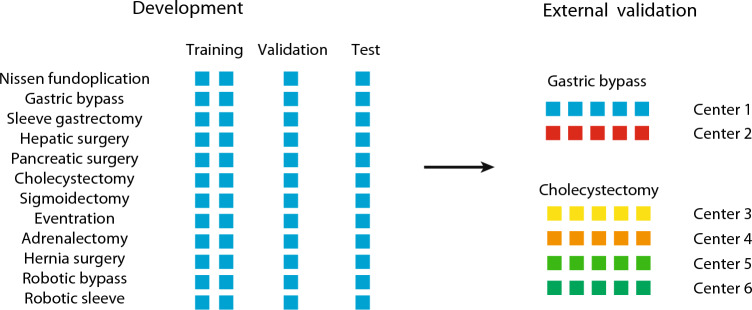
Illustration of dataset splits for model development, internal and external validation. Every square represents a video. Videos from the same center have the same color.

An illustration of the dataset split for model development, internal and multicentric external validation is displayed in Fig. 1.

Each hospital complied with local institutional review board (IRB) requirements. Patients either consented to the recording of their intervention or to the use of their health record for research purposes. All videos were shared as raw video material without identifying metadata. Therefore, the need for ethical approval was waived, except for Inselspital, Bern University Hospital, Switzerland where ethical approval was granted by the local IRB (KEK Bern 2021-01666).

### Annotations

Each video was split into frames at the rate of 1 frame per second. All frames were annotated in a binary fashion, being either inside the patient’s abdomen or out-of-body. The valve of the trocar was the visual cue for the transition from inside to out-of-body. All frames in which the valve of the optic trocar is visible are considered out-of-body to err on the safe side of privacy preservation. All datasets were annotated by a single annotator (A.V.). Edge cases were reviewed by a board-certified surgeon with extensive experience in surgical video analysis (J.L.L.).

### Model architecture and training

**Figure 2:**
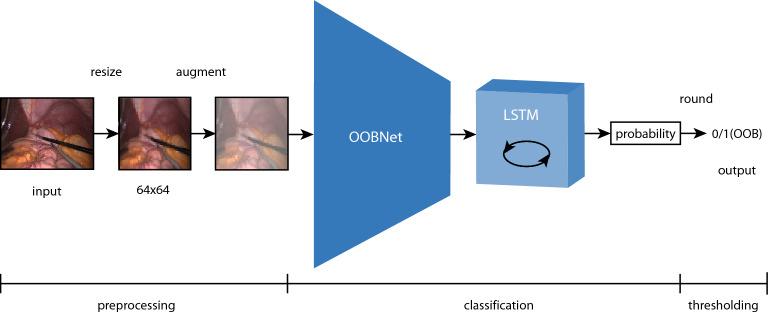
Model architecture of OoBNet. The input image is resized to 64 × 64 pixels and augmented with random rotation and contrast. Then it is fed to the deep neural network with a consecutive long short-term memory (LSTM) which outputs a probability like value whether the image is out-of-body or not. This probability is rounded at a 0.5 threshold to either 0 (inside the body) or 1 (out-of-body OOB).

OoBNet is a deep learning-based image classifier, using MobileNetV2^21^ as backbone followed by dropout (with dropout rate 0.5), a long short-term memory (LSTM with 640 units)^22^, linear and sigmoid layers. Layer normalization was applied before dropout and linear layers. MobileNetV2 is a model architecture designed for image recognition at low computational resources as in mobile devices and smartphones. The LSTM layer contains memory gates that bring context awareness to frame classification. As part of preprocessing, input images were resized to 64 × 64 pixels, then augmented with random rotation and contrast. Data augmentation is a common way to generate variance in the input dataset to improve the robustness of the model. The output of OoBNet is a probability-like value that is then binarized to either 0 or 1 to predict whether the image is an inside or out-of-body frame (Fig. 2).

The network was trained on video clips of 2048 consecutive frames for 300 epochs (cycles) with early stopping applied according to the highest F1-score obtained on the validation dataset. The optimizer used was Adam^23^ with a learning rate of 0.00009 and a batch size of 2048. The trained model and an executable application of OoBNet are available at https://github.com/CAMMA-public/out-of-body-detector.

### Model evaluation

OoBNet was evaluated on the test dataset, which was used neither for model training nor validation. Furthermore, external evaluation was done on two independent and multicentric datasets as described above. The predictions of OoBNet were compared to human ground truth annotations. The performance of OoBNet was measured as precision, recall, F1-score, average precision, and receiver operating characteristic area under the curve (ROC AUC). Precision is the proportion of true positives among all positive predictions (true and false positives), also referred to as positive predictive value. Recall is the proportion of true positives among all relevant predictions (true positives and false negatives), also referred to as sensitivity. F1-score is the harmonic mean of precision and recall. Average precision is the area under the precision-recall curve. ROC AUC is the area under the receiver operating characteristic curve which is created by plotting sensitivity against 1-specificity. It is also referred to as c-statistic.

## Results

OoBNet was trained, validated, and tested on an internal dataset of 48 videos with a mean duration ± standard deviation (SD) of 123 ± 79 min. containing a total of 356,267 frames. Thereof, 112,254 (31.51%) were out-of-body frames. External validation of OoBNet was performed on a gastric bypass dataset of 10 videos with a mean duration ± SD of 90 ± 27 min. containing a total of 54,385 frames (4.15% out-of-body frames) and on a cholecystectomy dataset of 20 videos with a mean duration ± SD of 48 ± 22 min. containing a total of 58,349 frames (8.65% out-of-body frames). The full dataset statistics and the distribution of frames across training, validation and test set are displayed in Table 1**.**

**Table 1:** Dataset statistics.

Dataset	Videos	Min. duration	Max. duration	Average duration	Total frames	Out-of-body frames	
	(n)	(min.)	(min.)	(min.)	(n)	(n)	(%)
Training	24	35	302	112	161,870	57,203	35.34
Validation	12	26	408	132	95,157	23,388	24.58
Test	12	33	239	137	99,240	31,663	31.91
Gastric bypass	10	55	134	90	54,385	2259	4.15
Center 1	5	66	134	101	30,464	696	2.28
Center 2	5	55	117	79	23,921	1563	6.53
Cholecystectomy	20	17	88	48	58,349	5050	8.65
Center 3	5	31	88	55	16,786	2268	13.51
Center 4	5	24	88	46	13,997	885	6.32
Center 5	5	44	76	57	17,371	356	2.05
Center 6	5	17	53	34	10,195	1541	15.12

**Figure 3:**
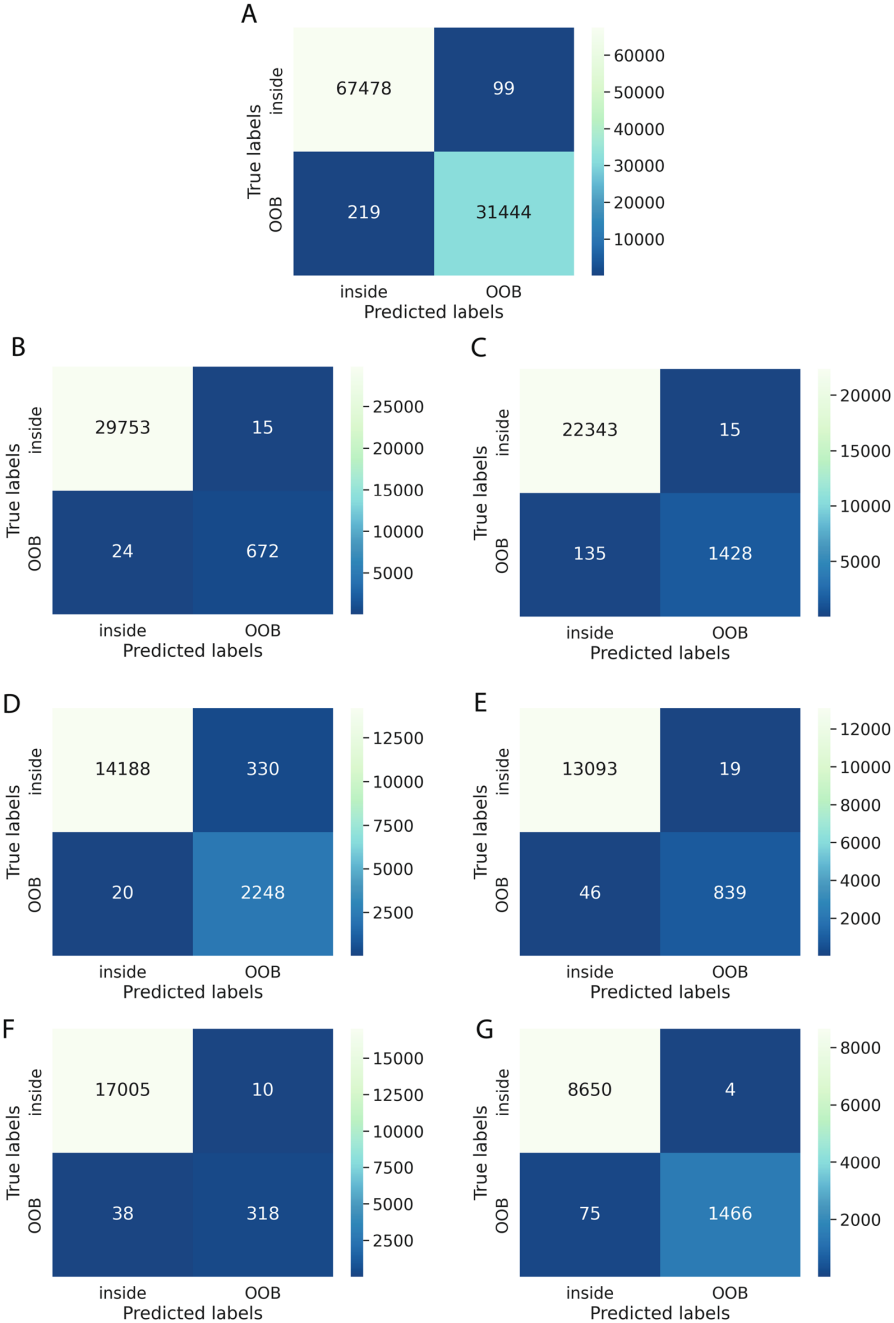
Confusion matrices. (**A**) Test set; (**B**) and (**C**) centers 1 and 2 (multicentric gastric bypass dataset); (**D**–**G**) centers 3, 4, 5, and 6 (multicentric cholecystectomy dataset).

**Figure 4:**
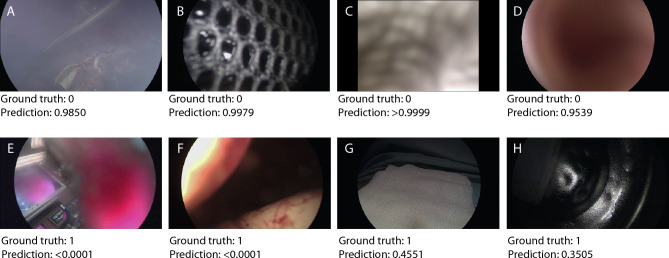
Qualitative results. Top row: False positive model predictions (OoBNet predicts the frame to be out-of-body even though it is not). Bottom row: False negative model predictions (OoBNet predicts the frame to be inside the body even though it is out-of-body). Below each image the binary human ground truth annotations and the probability like model predictions are provided. In (**A**), surgical smoke is impairing the vision. In (**B**–**D**), a mesh, a swab, and tissue are so close, that—lacking the temporal context—it is difficult to distinguish even for a human annotator whether it is out-of-body or not. In (**E**) and (**F**), blood on the endoscope and a glove with bloodstains mimic an inside view. In (**G**), a surgical towel covers most of the patient’s body, so that the model lacks visual cues for an out-of-body frame. In (**H**), the endoscope is cleaned in a thermos, which mimics the inside of a metal trocar.

The ROC AUC of OoBNet evaluated on the test set was 99.97%. Mean ROC AUC ± SD of OoBNet evaluated on the multicentric gastric bypass dataset was 99.94 ± 0.07%. Mean ROC AUC ± SD of OoBNet evaluated on the multicentric cholecystectomy dataset was 99.71 ± 0.40%. The full quantitative results are shown in Table 2. Confusion matrices on the test set, the multicentric gastric bypass dataset, and the multicentric cholecystectomy dataset are displayed in Fig. 3A–G. OoBNet was evaluated on a total of 111,974 frames, whereof 557 frames (0.50%) were falsely classified as inside the body even though they were out-of-body frames (false negative predictions). Qualitative results illustrating false positive and false negative predictions of OoBNet are displayed in Fig. 4. A video with qualitative results of OoBNet is provided in the Supplementary (Supplementary Video S1, which illustrates how endoscopic videos can be anonymized using OoBNet).

**Table 2:** Quantitative evaluation results on internal and external datasets.

Dataset	Videos (n)	ROC AUC (%)	Average precision (%)	F1-score (%)	Precision (%)	Recall (%)
Test	12	99.97	99.94	99.50	99.69	99.31
Gastric bypass^a^	10	99.94 ± 0.07	99.42 ± 0.26	96.10 ± 1.53	98.39 ± 0.81	93.96 ± 3.67
Center 1	5	99.99	99.60	97.18	97.82	96.55
Center 2	5	99.89	99.23	95.01	98.96	91.36
Cholecystectomy^a^	20	99.71 ± 0.40	98.66 ± 1.27	94.74 ± 2.17	95.43 ± 5.62	94.37 ± 4.01
Center 3	5	99.83	99.00	92.78	87.20	99.12
Center 4	5	99.92	98.93	96.27	97.79	94.80
Center 5	5	99.12	96.85	92.98	96.95	89.33
Center 6	5	99.97	99.85	96.93	99.79	94.22

^a^Results are mean ± standard deviation.

ROC AUC, receiver operating characteristic area under the curve.

## Discussion

This study reports the development and multicentric validation of a deep learning-based image classifier to detect out-of-body frames in endoscopic videos. OoBNet showed a performance of 99% ROC AUC in validation on three independent datasets. Using the provided trained model or the executable application, OoBNet can easily be deployed to anonymize endoscopic videos in a retrospective fashion. This allows to create video databases while preserving the patient’s and OR staff’s privacy and furthermore facilitates the use of endoscopic videos for educational or research purposes without revealing any sensitive information.

To our knowledge, OoBNet is the first out-of-body image classifier trained on videos of multiple interventions and validated on two external datasets. Previous work by our group used an unsupervised computer vision approach to identify out-of-body frames. Based on the redness- and brightness-levels of images, they were classified at an empirically set threshold as inside the body or out-of-body^24^. Zohar et al. used a semi-supervised machine learning approach to detect out-of-body scenes in a large dataset of laparoscopic cholecystectomy videos yielding a 97% accuracy^25^. However, this previous study has two major limitations. On the one hand, the main performance metric reported is accuracy. Accuracy is sensitive to the data distribution, or the prevalence of a given observation. On the other hand, it was trained on a dataset of a single intervention type only. This does not ensure that the model generalizes to other intervention types.

Typically, image classifiers are trained to distinguish visually distinct classes. Classifying images of an endoscopic video as inside or out-of-body seems analogous. However, between inside and out-of-body there is a transition where the camera is moved inside or outside the body which might appear ambiguous. Therefore, the definition when an image is inside or out-of-body is crucial. We defined that the valve of the optic trocar is the visual cue for the transition from inside to out-of-body and vice versa. To err on the side of privacy protection as soon as the valve is visible the frame is considered as out-of-body even if the camera is still inside the optic trocar. Using a LSTM module in the model architecture we took in to account the temporal context of inside and out-of-body frames and avoided misclassification in the phase transition from inside to out-of-body and vice versa due to phase flickering.

Despite OoBNet’s excellent performance, even in external validation, the model has its limitations. Not every frame was correctly classified. The ideal classifier would have neither false positive (predicted as out-of-body by the model though inside the body) nor false negative predictions (predicted as inside the body by the model though out-of-body). However, to err on the privacy preserving site, false negative predictions must be minimized. In other words, the threshold of the classifier needs to be optimized for sensitivity (recall). But maximum sensitivity and no false negatives predictions only can be achieved if every frame is classified as out-of-body. Though, this would be a completely unspecific classifier leading to a complete loss of the inside of body frames, which are relevant for surgical video analysis. Therefore, a tradeoff between precision and recall needs to be done. As F1-score is the harmonic mean of precision and recall, a classifier at maximum F1-score optimizes precision and recall at the same time. In this study the maximum F1-score on the validation set was used as early stopping criteria for model training and was achieved at a classifier threshold of 0.73. But as this threshold yielded more false negative predictions in favor of less false positive predictions, we used the default threshold of 0.5. Of note, the classifier threshold in this study was not learned by model training but manually set to minimize false negative predictions at an acceptable false positive rate. Using a threshold < 0.5 would have further reduced the number of false negatives, however, at an increased number of false positives (see the number of false negative vs. false positive predictions at different thresholds for all the three test sets Supplementary Fig. S1).

As qualitative results show (Fig. 4), the performance of OoBNet was limited if the endoscopic vision was impaired by surgical smoke, fog, or blood. Furthermore, OoBNet predicted false positive results when objects (mesh, swabs, tissue) were so close to the camera, that the vision was blurred, and even for a human annotator it was difficult to distinguish whether a given frame is out-of-body or not. Further work to improve OoBNet’s performance would include a model training on a larger set of those edge cases with impaired endoscopic vision. Moreover, OoBNet predicted false negative results if an out-of-body frame visually resembled an inside scene. Manual inspection of all false negative predictions (n = 557) on all test datasets revealed three privacy sensitive frames, where OR staff potentially could have been identified. However, out of 111,974 frames OoBNet was evaluated on not a single frame revealed the identity of the patient, the time, or the date of the intervention. Nevertheless, videos anonymized with OoBNet need manual revision to ensure medical confidentiality before they are stored, shared, or published. Though, OoBNet reduces the time needed for manual revision as false negative predictions are often situated in temporal proximity to true positive predictions.

In external validation OoBNet showed a drop of up to 6.7% points F1-score. This is in line with results from multicentric validation of other AI models in the surgical domain. For example, state of the art surgical phase recognition models have demonstrated variable performance in multicentric validation^26,27^. Furthermore, EndoDigest, a computer vision platform for video documentation of CVS in laparoscopic cholecystectomy, showed a 64–79% successful CVS documentation when validated on a multicentric external dataset compared to 91% successful CVS documentation on the internal dataset^14,20^. Therefore, the performance of AI models trained and evaluated on a single dataset should be regarded cautiously, and these results further highlight the need for external validation of AI models. Our model, however, has shown to generalize well on videos from several external centers.

The significance of OoBNet lies in its high reliability to identify of out-of-body frames in endoscopic videos. OoBNet is trained on a set of highly diverse endoscopic surgeries, including robotic surgeries, to account for the different visual appearances of anatomy, instruments and ORs. Furthermore, OoBNet is evaluated on two independent datasets to show its ability to generalize across centers. OoBNet is publicly shared as a tool to facilitate privacy preserving storing, handling, and publishing of endoscopic videos.

In conclusion, OoBNet can identify out-of-body frames in endoscopic videos of our datasets with a 99% ROC AUC. It has been extensively validated on internal and external multicentric datasets. OoBNet can be used with high reliability to anonymize endoscopic videos for archiving, research, and education.

## Supplementary Information

Supplementary Information 1.

Supplementary Video 1.

## Data Availability

The code of the model, the trained model weights and an executable are available at https://github.com/CAMMA-public/out-of-body-detector. Due to privacy restrictions the datasets used in the present work cannot be publicly shared.
