# Supplementary material for: Preserving privacy in surgical video analysis using a deep learning classifier to identify out-of-body scenes in endoscopic videos
Source: Sci Rep. 2023 Jun 7;13:9235. doi: 10.1038/s41598-023-36453-1 (PMC10247775; doi:10.1038/s41598-023-36453-1)

Supplementary Material

Preserving Privacy in Surgical Video Analysis Using a Deep Learning Classifier to Identify Out-of-Body Scenes in Endoscopic Videos

Joël L. Lavanchy, MD[1,2,6*#], Armine Vardazaryan, MSc[1,3#], Pietro Mascagni, MD PhD[1,4], AI4SafeChole Consortium[7], Didier Mutter, MD PhD[1,5], Nicolas Padoy, PhD[1,3]

1 IHU Strasbourg, France
2 Department of Visceral Surgery and Medicine, Inselspital, Bern University Hospital, University of Bern, Switzerland
3 ICube, University of Strasbourg, CNRS, France
4 Fondazione Policlinico Universitario Agostino Gemelli IRCCS, Rome, Italy
5 University Hospital of Strasbourg, France
6 Division of Surgery, Clarunis – University Center for Gastrointestinal and Liver Diseases, St Clara and University Hospital of Basel, Switzerland
7 Reprented by Bernard Dallemagne, Institute for Research Against Digestive Cancer (IRCAD), Strasbourg, France
# Joël Lavanchy and Armine Vardazaryan contributed equally and share first co-authorship

* Correspondence and requests for reprints to:
Joël L. Lavanchy, MD
IHU Strasbourg
1 Place de l’Hôpital
67091 Strasbourg Cedex, France
joel.lavanchy@ihu-strasbourg.eu

The code of the model, the trained model weights and an executable are available at https://github.com/CAMMA-public/out-of-body-detector.

Supplementary Video S1: The Video illustrates how endoscopic videos can be anonymized using Out-of-Body Network.

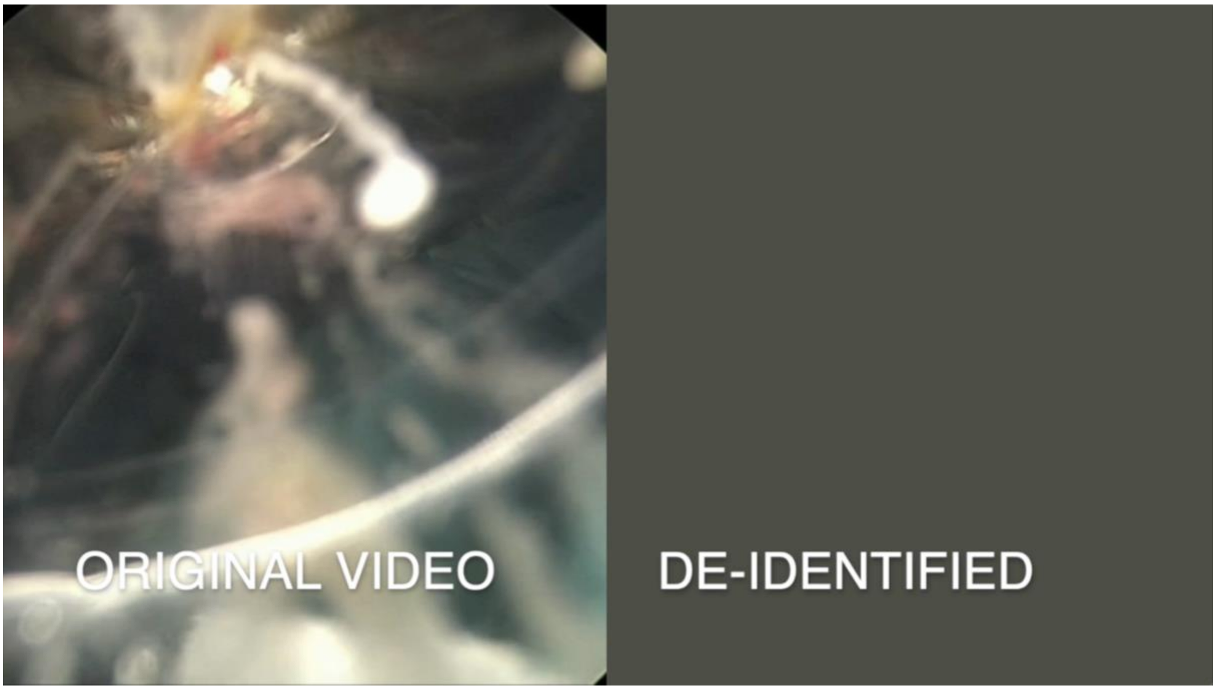

(OOB_Supplementary_Video1.mp4)

Supplementary Figure S1: Number of false negative (FN) and false positive (FP) predictions at different thresholds for A: test set; B: multicentric gastric bypass dataset; C: multicentric cholecystectomy dataset.

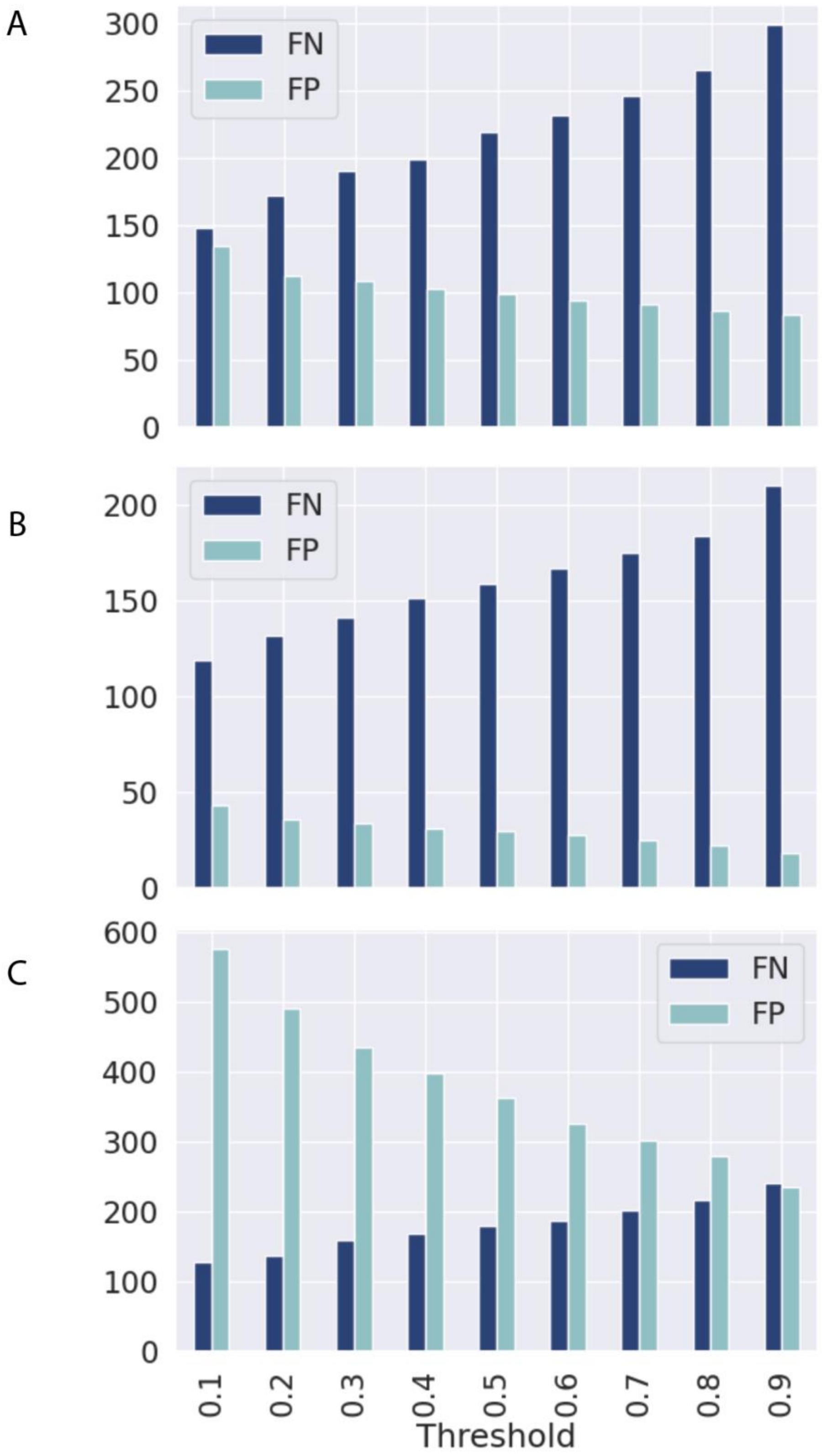

Supplement: Supplementary file 1 — Supplementary Information 1. [file 41598_2023_36453_MOESM1_ESM.pdf]
